# Genome Sequence of the Symbiotic Type Strain *Rhizobium tibeticum* CCBAU85039^T^

**DOI:** 10.1128/genomeA.01513-16

**Published:** 2017-01-26

**Authors:** Gonzalo Torres Tejerizo, Daniel Wibberg, Anika Winkler, Ernesto Ormeño-Orrillo, Esperanza Martínez-Romero, Karsten Niehaus, Alfred Pühler, Jörn Kalinowski, Antonio Lagares, Andreas Schlüter, Mariano Pistorio

**Affiliations:** a CeBiTec, Bielefeld University, Genome Research of Industrial Microorganisms, Bielefeld, Germany; b IBBM (Instituto de Biotecnología y Biología Molecular), CCT-CONICET-La Plata, Departamento de Ciencias Biológicas, Facultad de Ciencias Exactas, Universidad Nacional de La Plata, La Plata, Argentina; c Laboratorio de Ecología Microbiana y Biotecnología, Departamento de Biología, Facultad de Ciencias, Universidad Nacional Agraria La Molina, Lima, Peru; d Centro de Ciencias Genómicas, Universidad Nacional Autónoma de México, Cuernavaca, Morelos, Mexico

## Abstract

*Rhizobium tibeticum* was originally isolated from root nodules of *Trigonella archiducis-nicolai* grown in Tibet, China. This species is also able to nodulate *Medicago sativa* and *Phaseolus vulgaris*. The whole-genome sequence of the type strain, *R. tibeticum* CCBAU85039^T^, is reported in this study.

## GENOME ANNOUNCEMENT

Availability of nitrogen in soils is one of the main concerns for crop cultivation in agriculture. Nitrogen is essential for the biosynthesis of proteins, amino acids, vitamins, and other compounds. Chemical fertilizers commonly ensure sufficient nitrogen availability for crop production. However, these compounds may also cause environmental problems (1, 2). Alternatively, certain bacteria and archaea are able to fix atmospheric dinitrogen via reduction to ammonia. Rhizobia belonging to the classes *Alphaproteobacteria* or *Betaproteobacteria* inhabit soils and are able to enter into nitrogen-fixing symbiosis with leguminous plants. They induce the development of root nodules, where, after colonization and differentiation, bacteroids are able to fix dinitrogen (3). Rhizobia are highly diverse regarding their genetic, metabolic, and taxonomic characteristics (4). *Mesorhizobium loti* MAFF303099 (5) and *Ensifer meliloti* 1021 (6) were the first rhizobia for which complete genome sequences became available. Recently, the genome sequences of 163 further root-nodule bacteria were published, extending our knowledge regarding this group of bacteria (7). However, particular and important species/groups were not covered by the study cited above. An important clade within the group of rhizobia comprises the strains *Rhizobium mesoamericanum* CCG502^T^ (8)*, Rhizobium grahamii* CCG501^T ^(9), *Rhizobium favelukesii* LPU83^T^ (10), and *Rhizobium tibeticum* CCBAU85039^T^ (11). While genome sequence information is available for the first three strains, the genome of *R. tibeticum* CCBAU85039^T^ had not yet been sequenced. The latter strain is able to nodulate *Phaseolus vulgaris*, *Medicago lupulina*, *Medicago sativa*, *Trigonella archiducis-nicolai*, and *Trigonella foenum-graecum*. With the objective to uncover mechanisms of rhizobial diversification and to complement genome sequence information for rhizobial species, the *R. tibeticum* CCBAU85039^T^ genome was sequenced.

Genomic DNA of *R. tibeticum* CCBAU85039^T^ was isolated using the GENTRA Pure-Gene kit (Qiagen). A sequencing library was constructed and sequenced on the MiSeq platform applying the Illumina paired-end protocol (Illumina, Inc.). In total, 4,090,570 sequence reads were obtained, yielding a total of 1,102,161,679 bp of sequence information. The Illumina reads were assembled by the GS *de novo* assembler software (gsAssembler, version 2.8; Roche) with a final outcome of 206 large (>500 bp) contigs. Of these, 167 contigs were arranged in 128 scaffolds. The estimated genome size is around 7 Mb, and, accordingly, the coverage obtained was approximately 159-fold. The genome features an average G+C content of 59.72%. The *N*_50_ value for scaffolds was 177,123 bp with an average scaffold size of 54,157 bp.

The genome was annotated applying the Prokka pipeline and GenDB (12, 13), which predicted 6,977 protein-coding sequences (CDSs) and 45 tRNA genes. The rRNA operon was found on a 4-fold overrepresented contig, suggesting the presence of four *rrn* copies within the genome. Genome comparisons were done within the EDGAR version 2.0 platform (14). More than 5,200 CDSs of *R. tibeticum* (75% of all CDSs) represent orthologs to corresponding *R. favelukesii* LPU83^T^ genes (15). Moreover, phylogenetic analysis of the concatenated core genomes confirmed a close relationship between both strains, as previously described (16). Further comparative studies will elucidate the similarities and differences among different groups of sequenced rhizobial strains and refine their taxonomic classification.

### Accession number(s).

This whole-genome shotgun project has been deposited in the EMBL database under the accession numbers FNXB01000001 to FNXB01000167. The version described in this paper is the first version, FNXB01000000.
